# Decreased proliferation of human melanoma cell lines caused by antisense RNA against translation factor eIF-4A1

**DOI:** 10.1038/sj.bjc.6600351

**Published:** 2002-06-17

**Authors:** J Eberle, L F Fecker, J-U Bittner, C E Orfanos, C C Geilen

**Affiliations:** Department of Dermatology, University Medical Center Benjamin Franklin, The Free University of Berlin, Fabeckstrasse 60-62, 14195 Berlin, Germany

**Keywords:** melanoma, translation control, antisense, proliferation, apoptosis

## Abstract

Control of translation initiation was recognised as a critical checkpoint for cell proliferation and tumorigenesis. In human melanoma cells, we have previously reported consistent overexpression of translation initiation factor eIF-4A1. Here, we investigated by transfection of antisense constructs its significance for the control of melanoma cell growth. The tetracycline-inducible expression system was established in melanoma cells, and three fragments of the 5′-, central-, and 3′-portion of the eIF-4A1 cDNA were subcloned in antisense and in sense orientation after a tetracycline inducible promoter. Significant proliferation decrease was obtained after transient transfection and induction of antisense RNA directed against the 5′- and the central portion (up to 10%), whereas, no effects were seen after induction of the 3′-fragment and the sense controls. Cell clones stably transfected with the central antisense fragment revealed after doxycycline induction reduced expression of endogeneous eIF-4A1 mRNA correlated with decreased proliferation rates (up to 6%). These data demonstrate the applicability of antisense strategies against translation factors in melanoma cells. Translation initiation factor eIF-4A1 contributes to the control of melanoma cell proliferation and may be taken into consideration when scheduling new therapeutic approaches targeting the translational control.

*British Journal of Cancer* (2002) **86**, 1957–1962. doi:10.1038/sj.bjc.6600351 www.bjcancer.com

© 2002 Cancer Research UK

## 

Control of protein synthesis is known as a cellular mechanism important for saving energy resources and for rapidly responding to changing growth requirements. In recent years more and more data are emerging on the involvement of this process in cell growth and in tumorigenesis. It is mainly the initiation of translation, which turned out to be a checkpoint for cell proliferation.

In eucaryotes, initiation of translation, which brings mRNA and ribosomal subunits together, is triggered by a number of initiation factors (eIF). This process may be subdivided into five steps corresponding to five groups of translation initiation factors (Rhoads, 1993). The starting point of the initiation process may be seen in the binding of eIF-2 to the initiator-tRNA and to GTP in a ternary complex, which subsequently binds the 40S small ribosomal subunit resulting in the 43S pre-initiation complex. In parallel, eIF-4E binds to the mRNA cap structure as well as to eIF-4G, which itself is a large polypeptide supplying binding sites for eIF-4A and eIF-3. This complex enables transient melting of the mRNA secondary structure to then allow the ribosomal subunit to enter and scan the mRNA in 3′ direction for the initiation codon. For RNA melting, the helicase activity described for eIF-4A is essential (Ray *et al*, 1985).

In current therapeutic strategies against cancer less interest has been focussed on protein synthesis mechanisms. However, an increasing body of data is emerging about the involvement of translational processes and factors in control of cell proliferation, also indicating that protein synthesis can be an additional target for anticancer strategies (Caraglia *et al*, 2000). In order to characterise malignant melanoma cells, we had isolated and identified a number of genes specifically regulated in human melanoma cells as compared to normal human melanocytes (Eberle *et al*, 1995a,b, 1997, 1999). Within this group of genes, we identified the translation initiation factor eIF-4A1 (Eberle *et al*, 1997), found consistently upregulated at the mRNA level. As the oncogenic potential of other translation initiation factors (e.g., eIF-4E and eIF-2, had already been described in other cells; Lazaris-Karatzas *et al*, 1990; Donze *et al*, 1995), we suggested that eIF-4A1 upregulation may contribute specifically to the malignant phenotype of melanoma. In the present paper, we addressed the question whether we can confer inhibitory effects on melanoma cells by obstructing eIF-4A1 gene expression using transient and stable transfection of antisense constructs against eIF-4A1.

## MATERIALS AND METHODS

### Cell cultivation and transfection

Melanoma cell lines were maintained in DMEM (4.5 g l^−1^ glucose; Gibco, Paisley, Scotland) supplemented with 10% foetal calf serum, penicillin and streptomycin (Biochrom, Berlin, Germany). For cell transfection, eight different lipids were tested (Pfx-1–Pfx-8; Invitrogen, Groningen, Netherlands), and for the subsequent experiments, Pfx-2, which showed the best transfection efficiency was consequently used. At a confluence of 50%, cells were washed in serum-free Opti-MEM (Gibco) and were incubated at 37°C in the same medium containing 0.5% Pfx-2 and 1.5 μg ml^−1^ plasmid DNA. After 2 h, cells were washed once and were further incubated with standard growth medium, which contained 2 μg ml^−1^ doxycycline (ICN, Aurora, OH, USA) in case of induction. For stable transfections, selective antibiotics, geneticin (Gibco; 400 μg ml^−1^) or hygromycin (Boehringer, Mannheim, Germany; 100 μg ml^−1^), were added, and selection was carried out until only transfected cells remained. Cell clones were obtained by limited dilution in microtitre plates and were continuously cultured under antibiotic pressure (geneticin and/or hygromycin).

Two tetracycline-inducible cell lines SKM13-Tet-On and Bro-Tet-On were established from the cell lines Bro and SK-Mel-13 (Carey *et al*, 1976; Lockshin *et al*, 1985), respectively, by stable transfection of the pTet-On plasmid (Clontech, Palo Alto, CA, USA). The tetracycline-inducible gene expression system had been described by Gossen and Bujard (1992). After stable transfection, individual cell clones were screened for their tetracycline inducibility by transient transfection of the luciferase-encoding plasmid pTRE-Luc (Clontech) followed by a luciferase assay (De Wet *et al*, 1987; Promega, Madison, WI, USA), 48 h after transfection. For promoter induction, doxycycline was used instead of tetracycline as recommended by the supplier of the vector system (Clontech). As a standard for the luciferase assay, purified luciferase (Promega; 3.3×10^10^ light units per mg protein) was used. Double stable transfectants were obtained after transfection of Bro-Tet-On with an eIF-4A1 antisense construct in pTRE-1 and co-transfection with the hygromycin resistance plasmid pTK-Hyg (1 : 20 molar ratio; both plasmids from Clontech).

### Construction of antisense and control plasmids

Of the eIF-4A1 cDNA, which we had cloned earlier (Eberle *et al*, 1997), three different fragments from the 5′ end, of the central portion and from the 3′ end, respectively, were subcloned by restriction and ligation in antisense and in sense orientation, in one or two copies, behind the tetracycline-inducible CMV promoter present in the plasmid pTRE-1. Plasmid DNA used for cell transfection was purified by an endotoxin-free plasmid purification kit (Birnboim and Doly, 1979; Qiagen, Hilden, Germany).

### RNA extraction and Northern blotting

Cells were harvested at 70% confluence, after adding fresh growth medium 24 h before. Total RNA was isolated from trypsinised cells using the RNeasy Mini Kit (MacDonald *et al*, 1987; Qiagen, Hilden, Germany). For Northern blotting (Thomas, 1980), 10 μg of total RNA per lane were separated in formaldehyde/agarose gels (1.2% agarose). For determination of mRNA length, a 0.24–9.5 kb RNA ladder (Gibco, Gaithersburg, TN, USA) was used. RNA was transferred by capillary blotting to Gene Screen Plus membranes (NEN Life Science, Cologne, Germany), and hybridisations were carried out at 60°C for 16 h. Probes for eIF-4A1 and β-actin derived from a differential screening (Eberle *et al*, 1995a); the cDNA fragments were radiolabelled by random priming (Gibco) followed by purification with Nuc-trap push columns (Stratagene, La Jolla, CA, USA). Membranes were washed under stringent conditions (45 min at 65°C in 2× sodium citrate buffer, SSC) and were then exposed to an imaging plate for quantification of radioactivity by a BAS-1500 Phosphoimager (Fuji, Nakanuma, Japan). For reasons of normalisation, membranes were boiled in water and rehybridised with a probe for β-actin. Quantified individual expression values were first normalised by corresponding β-actin values and then compared with each other.

### Determination of cell proliferation and cell number

Cell proliferation was determined by the thymidine incorporation assay (O'Keefe *et al*, 1988). Cells were seeded in 96-well plates and were transiently transfected with a given plasmid construct (60 wells of a microtitre plate corresponded to one transfection experiment, wells of the margin were only filled with growth medium). For induction, doxycycline was added to every second well. Two days after transfection, wells were washed once with growth medium, and cells were incubated in growth medium containing ^3^H-thymidine (2.5 μCi ml^−1^; 68.5 Ci mmol^−1^) for 2 h. After harvesting the cells with a cell harvester, tritium incorporation was measured in a scintillation counter. Ratios of each two neighbouring wells (induced *versus* non-induced) were determined and a mean value (induced *versus* non-induced) was calculated for each microtitre plate. At least three independent experiments (three microtitre plates) were performed for each combination of cell line and plasmid construct, and finally mean values and standard deviations were calculated from individual experiments.

Comparison of proliferation of stably transfected cell clones was performed in a similar way, only no transient transfection was needed, and cells were harvested 2 days after induction with doxycycline. Also here, one experiment corresponded to one microtiter plate and at least three independent experiments were performed for each cell line.

Cell numbers were determined 2 days after transient transfection either by direct cell counting in a Neubauer chamber or by the crystal violet assay described by Gillies *et al* (1986). In brief, cells grown in six-wells were fixed for 30 min with 1% glutaraldehyde and stained for another 30 min in a 0.01% crystal violet solution in PBS. Plates were washed in deionised water for 30 min, and were then completely dried. Crystal violet absorbed by cell nuclei was solubilised with 0.2% Triton X-100 in PBS. Aliquots were measured in an ELISA-Reader at 550 nm.

### Statistical analysis

For statistical analysis, the individual values (single wells) of an experiment were normalised by the mean value of the respective experiment. Thus, values of independent experiments could be combined in groups and resulted in an induced (+) group and a not induced (−) group. To demonstrate statistical significance of inhibition of cell proliferation or reduced cell numbers due to induction of antisense constructs, Student's *t*-test was applied (unpaired and heteroscedastic).

## RESULTS

### Establishment of Tet-On melanoma cell lines

As a prerequisite for applying the tetracycline-regulated gene expression system described by Gossen and Bujard (1992) for melanoma cells, we prepared suitable Tet-On cell lines by stable transfection of the pTet-On plasmid into the undifferentiated and amelanotic melanoma cell line Bro as well as into the differentiated melanoma cell line SK-Mel-13 characterised both by dendrite and melanin formation. After transfection, several geneticin-resistant cell clones were isolated, which were subsequently screened for their doxycycline inducibility using a luciferase reporter plasmid. One clone of each transfection was selected which showed the highest doxycycline inducibility: Bro-Tet-On and SKM13-Tet-On.

In order to optimise transient melanoma cell transfection, a series of different lipids and conditions were tested. The use of different lipids resulted in dramatic differences in transfection efficiency of more than 200-fold; responses of both melanoma cell lines to different lipids were in parallel (data not shown). Thus, optimisation of transfection conditions for a certain cell type seemed to be absolutely required. Luciferase activity obtained by the improved transfection protocol (see Materials and Methods) and after doxycycline induction was equivalent to 7×10^6^ enzyme units per 10^6^ Bro-Tet-On cells, and to 3×10^6^ units per 10^6^ SKM13-Tet-On cells, respectively, what was more than 1000-fold over background (untransfected cells). The induction factors resulted from doxycycline treatment as compared to non-induced cell cultures were 22±6 (Bro-Tet-On) and 28±9 for SKM13-Tet-On, respectively.

### Construction of antisense plasmids

For expression of antisense-RNA directed against the translation initiation factor eIF-4A1, three different cDNA fragments, from the cDNA 5′-end, of the central portion and from the 3′-end, were subcloned in antisense orientation into a tetracycline responsive expression plasmid downstream of the tetracycline-inducible promoter. For answering the question whether a higher copy number may be critical for an antisense effect, the central-fragment was also subcloned in tandem in antisense orientation. As negative controls, the central-fragment was subcloned as one and as two tandem copies in sense orientation (Table 1

**Table 1:**
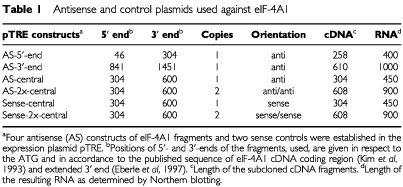
Antisense and control plasmids used against eIF-4A1

).

**Figure 1:**
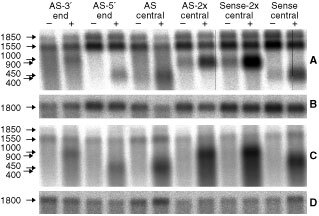
Expression of antisense RNA. Northern blots are shown of the cell lines SKM13-Tet-On (**A** and **B**) and of Bro-Tet-On (**C** and **D**). Total RNA was extracted 2 days after transient transfection of six different antisense and sense constructs for translation initiation factor eIF-4A1 subcloned behind a tetracycline-inducible promoter (names of constructs are given in the top). Cell cultures grown with doxycycline (+) were compared with cultures grown without doxycycline (−). Northern blots were hybridised with cDNA probes specific for eIF-4A1 (**A** and **C**) and for β-actin (**B** and **D**), respectively. The position and length of the endogeneous mRNAs for eIF-4A1 (1850 N and 1550 N), of β-actin (1800 N), and of the different plasmid-derived RNAs is indicated at the left margin.

For investigating the expression and inducibility of corresponding RNAs, cells were transiently transfected with the plasmid constructs, and doxycycline induced cells were compared 2 days later with non-induced cells, at the RNA level. As demonstrated in Figure 1

, specific RNA expression was found after transient transfection for all plasmids constructed. After doxycycline induction, a strong increase of all plasmid-encoded RNAs was visible in both cell lines when compared to cultures grown without doxycycline. The steady state concentrations of endogeneous eIF-4A1 mRNA were difficult to quantify due to the high background of construct-specific RNA after transient transfection, but were not significantly reduced for any of the constructs, two days after transfection and induction (data not shown).

### Antiproliferative effects caused by antisense constructs against eIF-4A1, after transient transfection and induction of antisense constructs

**Figure 2:**
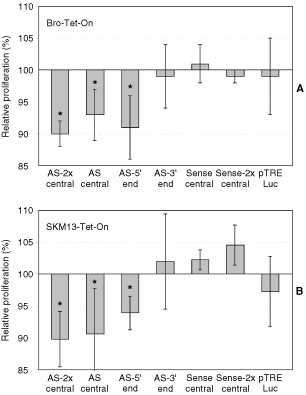
Reduced proliferation after transient transfection. Cell lines Bro-Tet-On (**A**) and SKM13-Tet-On (**B**) were transiently transfected with four antisense constructs (AS), two sense controls and the luciferase-encoding plasmid pTRE-Luc used as an additional control. Proliferation rates were determined in microtiter plates two days after transfection and induction with doxycycline. Relative proliferation rates were calculated as the ratio between cultures induced with doxycycline versus non-induced cultures (in %), and bars represent deviations from 100%. Each of the bars corresponds to mean values of at least three independent experiments of each 60 counted wells (180 wells). Significant reduction of proliferation is indicated by asterisks. According to Student's *t*-test, *P*-values for Bro-Tet-On were <0.0001 (AS-2x-central), <0.005 (AS-central), <0.0002 (AS-5′-end); *P*-values for SKM13-Tet-On were <0.0001 (AS-2x-central), <0.0001 (AS-central), <0.02 (AS-5′-end).

As translation efficiency may be a critical checkpoint for cell proliferation, we determined cell proliferation rates after transient transfection and compared cells induced with doxycycline *versus* cells grown without. As shown in Figure 2

, thymidine incorporation rates in cells transiently transfected with three antisense constructs were significantly reduced after doxycycline induction. Relative proliferation rates for induced Bro-Tet-On cells as compared to non-induced cells were at 90.4±2.5% after transfection of *antisense-2x-central*, 92.6±3.8% for *antisense-central*, and 90.8±4.6% for *antisense-5*′*-end* (Figure 2A). The antiproliferative antisense effects observed for SKM13-Tet-On after induction were largely in parallel: 89.8±4.3% for *antisense-2x-central*, 90.6±7.1% for *antisense-central*, and 93.9±2.6% for *antisense-5*′*-end* (Figure 2B). As each experiment consisted of 60 individually counted wells and was repeated at least three times, values were statistically significant (P-values see legend of Figure 2).

No antiproliferative effect, however, was seen for both of the sense controls (*sense-central* and *sense-2x-central*) as well as for a luciferase-encoding plasmid used as an additional control. Also, no antiproliferative effect was seen for the antisense construct directed against the 3′-end (*antisense-3*′*-end*) for any of both cell lines. As can be concluded from the comparison of the effects caused by *antisense-central* *versus* *antisense-2x-central*, doubling of the copy number was not able to significantly increase the antiproliferative effect (Figure 2).

Also, cell numbers were determined after transient transfection of AS-2x-central as compared to sense-2x-central in SKM13-Tet-On and Bro-Tet-On. Significantly reduced cell numbers were found 2 days after induction of antisense RNA, comparable to the results obtained by the tritium thymidine incorporation assay (Table 2

**Table 2:**
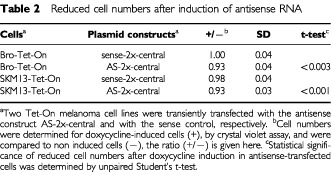
Reduced cell numbers after induction of antisense RNA

).

### Reduced proliferation in stable transfected melanoma cell lines after induction of antisense constructs

**Figure 3:**
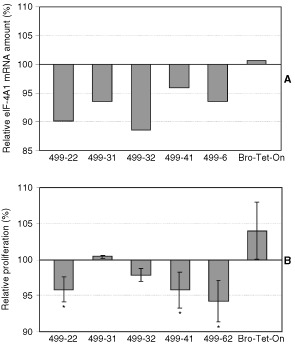
Reduced proliferation after induction of AS-2x-central in stably transfected cell clones. The cell line Bro-Tet-On was stably transfected with the antisense construct AS-2x-central. Five individual cell clones (499) as well as the parent cell line Bro-Tet-On were analysed 2 days after induction with doxycycline. (**A**) Amount of endogeneous mRNA for eIF-4A1, as determined by Northern blotting, densitometry and normalisation with β-actin mRNA (median values of two independent experiments). (**B**) Relative proliferation rates were calculated as the ratio between cultures induced with doxycycline *versus* non-induced cultures (in %), and the bars represent deviations from 100%. Each of the bars corresponds to mean values of at least three independent experiments of each 60 counted wells (180 wells). Significant reduction of proliferation is indicated by asterisks. According to Student's *t*-test, *P*-values were <0.03 (499-22), <0.02 (499-41), <0.002 (499-62).

To investigate whether the antiproliferative effect is retained after stable transfection, when smaller amounts of antisense-RNA have to be expected than after transient transfection, we stably transfected the melanoma cell line Bro-Tet-On with *antisense-2x-central*. In the following, five individual hygromycin-resistant cell clones were analysed. We first looked for eIF-4A1-specific RNA in the transfected cell clones, after doxycycline induction: No detectable levels of antisense RNA were found after Northern analysis, however, quantification of the endogeneous sense mRNA for eIF-4A1 (1850 N and 1550 N) revealed a decrease 2 days after doxycycline induction in all of the five clones, whereas the parent cell line, Bro-Tet-On, did not respond to doxycycline (Figures 3A

and 4

**Figure 4:**
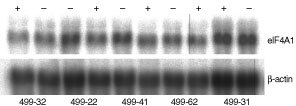
Reduced eIF-4A1 mRNA amounts after induction of AS-2x-central in stably transfected cell clones. The Tet-On cell line Bro-Tet-On was stably transfected with the antisense construct AS-2x-central. Amounts of eIF-4A1 mRNA were determined by Northern blotting after induction (+) and without induction (−) with doxycycline in five individual cell clones (499-). Expression levels for β-actin were analysed in parallel and were used for normalisation.

).

Proliferation assays revealed for three of the five clones significantly reduced proliferation rates after doxycycline induction as compared to non-induced cells, whereas, the parent cell line Bro-Tet-On did not show a proliferation decrease (Figure 3B).

## DISCUSSION

Gene expression in eucaryotes is regulated at several levels including the level of mRNA translation. In the last years, a series of reviews has been published on translational control giving a high impact on its role in cell growth and tumorigenesis (Clemens and Bommer, 1999; Jagus *et al*, 1999; Willis, 1999; Caraglia *et al*, 2000; Zimmer *et al*, 2000). Translational control no longer appears as a general and unselective control mechanism, but an increasing number of proteins were identified which are selectively regulated and which are involved in control of cell cycle and proliferation. Initiation of protein synthesis turned out as the rate-limiting step in translational control with particular attention being paid to expression and activation of eIF-2α and eIF-4E, which are activated and inactivated by phosphorylation, respectively (Jagus *et al*, 1999; Zimmer *et al*, 2000).

A relation between translational control and malignant cell transformation became obvious, when elevated levels of eIF-4E as well as reduced levels of the eIF-2α kinase, PKR, were detected in cancer cells (Kerekatte *et al*, 1995; Nathan *et al*, 1997; Jagus *et al*, 1999). Furthermore, overexpression of eIF-4E induced transformation (Rhoads, 1993), and its inhibition by antisense RNA decreased the malignancy of transformed cells (Graff *et al*, 1995). The oncogenic potential of eIF-2α was demonstrated by inhibition of its phosphorylation (Jagus *et al*, 1999). These findings on eIF-4E, eIF-2α and their kinases have resulted in the definition of a new group of *translational oncogenes*. Only limited data were available about the expression of translation factors in melanoma as well as about the role of eIF-4A1 in tumorigenesis. In a previous study, we reported consistent upregulation of eIF-4A1 mRNA in melanoma cell lines, as compared to normal human melanocytes, which led us suggest that eIF-4A1 may be related to melanoma cell transformation (Eberle *et al*, 1997).

Antisense techniques are powerful strategies to act against the malignant potential of genes upregulated in cancer. Having a pharmaceutical approach in mind, preferentially antisense oligonucleotides have been tested for their usefulness in cancer therapy. For an antisense-based, gene therapeutic approach, however, viral vectors and antisense RNA expression are needed. For melanoma, clinical studies using antisense oligonucleotides against the antiapoptotic Bcl-2 are on the way aiming the sensitisation of tumour cells against chemotherapy (Jansen *et al*, 2000). Several other genes were targeted in melanoma cells by *in vitro* studies, as the anti-apoptotic factor survivin (Grossman *et al*, 1999), the transcription factor c-*myc* (Citro *et al*, 1998) and the growth factor pleiotrophin (Satyamoorthy *et al*, 2000).

In the present study, we investigated whether eIF-4A1 can be targeted in cultured melanoma cells by overexpression of antisense RNA using antisense plasmid constructs. In order to reach high comparability, we established the tetracycline-regulated expression system in human melanoma cell lines, which allowed the direct comparison between induced and non-induced cells; also relatively weak differences could be quantified this way. Significant antiproliferative effects were found after induction of three different eIF-4A1 antisense constructs, both after transient and after stable transfection. The only limited antiproliferative effect which was obtained in all transfection experiments (up to 10% reduction) has to be seen in relation to the relatively high expression of eIF-4A1 mRNA in melanoma cells, comparable to the expression of major proteins as glycolytic enzymes and β-tubulin (Eberle *et al*, 1995a).

Due to the experience with antisense oligonucleotides, it is generally assumed that antisense can either result in reduction of the targeted mRNA due to specific ribonucleases or in direct obstruction of translation (Crooke, 1999). After stable transfection, we observed reduction of eIF-4A1 steady state RNA level, whereas this was not seen after transient transfection indicating that reduction of mRNA was not necessary in these experiments for obtaining the antiproliferative effect. Doubling of the copy number by using a tandem repeat of an antisense fragment did not further enhance the antisense effect, possibly indicating that a saturation point was already reached. Experience with antisense oligonucleotides had also shown that the effect of a given oligonucleotide is strongly dependent on the particular sequence targeted (Dean *et al*, 1994). This was also true in our approach, where we found comparable effects for a 5′ fragment and a central fragment whereas a 3′ fragment did not result in any proliferation effect. The data presented here demonstrate that human melanoma cells can be targeted by antisense constructs, even when the targeted mRNA is strongly expressed as in the case of eIF-4A1.

Due to a sequence comparison of translationally controlled mRNA species, it was predicted that many proliferation-related proteins will be regulated at the translational level (Kozak, 1991). The predicted secondary structures in their 5′ untranslated regions seem to be inhibitory for the ribosomal scanning process resulting in a dependence on the unwinding machinery (Sonenberg, 1996), which is based in particular on RNA helicase activity of eIF-4A1. There are many examples of translationally regulated growth factors and cytokines (Nielsen *et al*, 1995; Vagner *et al*, 1995; Bernstein *et al*, 1997). Furthermore, translational control has been shown for the cell cycle regulator cyclin D1 (Rosenwald *et al*, 1993), the transcription factor c-*myc* (Nanbru *et al*, 1997), several protein kinases (Marth *et al*, 1988; Yew *et al*, 1993) as well as for the key enzymes of polyamine synthesis which is closely linked to cell proliferation (Ruan *et al*, 1996). Therefore, the significance of translational control for cell proliferation became obvious. Furthermore, the oncogenic potential of several translationally regulated genes has been reported. In agreement, we found significant antiproliferative effects after induction of antisense RNA against eIF-4A1, strongly suggesting that in human melanoma cells, eIF-4A plays at least an auxiliary role in regulation of cellular proliferation. This assumption is further promoted by the finding that only eIF-4A1 but none of the other group 4 translation factors (eIF-4E, eIF-4B, eIF-4G, eIF-4A2) was significantly upregulated in melanoma cells as compared to normal melanocytes (Eberle *et al*, 1997).

In conclusion, our data underline the role of translation factors for cell proliferation and support the concept of translational oncogenes. Recent studies have focussed on the role of the factors eIF-4E and eIF-2α in malignant cell transformation; no data were available on melanoma cells. Our data suggest that a concerted action of several translation factors, including eIF-4A1, may be decisive for the translational contribution to melanoma development. It is likely that future therapeutic strategies will simultaneously target several genes also including the translational level. Due to the rather limited growth effect obtained after stable transfection, antisense gene therapies exclusively against eIF-4A1 would be not sufficient in melanoma, however, they may be contributory and helpful in combination with other gene targets.
